# Double blind control trial of vitamin D fortified milk on the expression of lncRNAs and adiponectin for patients with metabolic syndrome

**DOI:** 10.1186/s13098-023-00979-1

**Published:** 2023-01-18

**Authors:** Mohammad Rashidmayvan, Reza Sahebi, Amir Avan, Payam Sharifan, Habibollah Esmaily, Asma Afshari, Elyas Nattagh-Eshtivani, Fatemeh Najar Sedghdoust, Malihe Aghasizadeh, Gordon A. Ferns, Majid Ghayour-Mobarhan

**Affiliations:** 1 Department of Nutrition, Faculty of Medicine, Mashhad University of Medical Sciences, Mashhad, Iran; 2 Metabolic Syndrome Research Center, School of Medicine, Mashhad University of Medical Sciences, Mashhad, Iran; 3 Department of Biostatistics, School of Health, Mashhad University of Medical Sciences, Mashhad, Iran; 4 Iranian UNESCO Center of Excellence for Human Nutrition, Mashhad University of Medical Sciences, Mashhad, Iran; 5 Division of Medical Education, Brighton & Sussex Medical School, Falmer, Brighton, Sussex UK

**Keywords:** Adiponectin, Adiponectin antisense, LncRNA, MALAT1, Metabolic syndrome

## Abstract

**Background:**

Metabolic syndrome (Mets) is a common metabolic disorder in which hypoadiponectinemia is one of the consequences for the body caused by inflammation, and vitamin D may help improve inflammatory symptoms. LncRNAs (long non-coding RNA) play several different regulatory roles in the body. The goal of this study was to see how adding vitamin D to milk affected the levels of adiponectin and inflammatory lncRNAs in the serum of people with Mets.

**Methods:**

This clinical trial was conducted on staff and students between the ages of 30 and 50 at Mashhad University of Medical Sciences and met the International Diabetes Federation’s criteria for Mets. Eighty-two Mets were assigned randomly to one of two groups for ten weeks: fortified milk (FM) with 1500 IU vitamin D or non-fortified milk (NFM). Total RNA was extracted from both frozen clinical samples using Trizol reagent. APQ AS and MALAT1 lncRNA gene expression were measured by Real-Time PCR.

**Results:**

Serum adiponectin levels in the FM group increased significantly compared to the NFM group (p = 0.01). Also, the expression of APQ AS and MALAT1 genes decreased after ten weeks, which showed a significant decrease in APQ AS (p = 0.036).

**Conclusion:**

As in FM, vitamin D may have anti-inflammatory effects and increase adiponectin levels in people with Mets via decreasing APQ AS gene expression.

## Introduction

Mets is a common metabolic disorder defined by the WHO as a pathologic condition characterized by abdominal obesity, insulin resistance, hypertension, and hyperlipidemia [1]. The global prevalence of this disorder is estimated to be 14–32%, increasing with age in both sexes [2] and 33.7% in Iranian adults [3]. Epidemiological, genetic, and environmental factors such as diet play a key role in Mets development and progression [4]. Mets is a life-threatening condition caused by lifestyle and dietary changes that can be reduced by maintaining a healthy diet [5]. Obesity increases inflammatory cytokines and signaling in the body [6, 7]. The contribution of the Mets to the development of an inflammatory response is well documented [1].

Increased serum pro-inflammatory cytokines, including interleukin 6 (IL-6), resistin, tumor necrosis factor (TNF-α), and C-reactive protein (CRP), reflect overproduction by the expanded adipose tissue mass [8]. Adiponectin is an adipocyte-produced cytokine with anti-inflammatory properties related to insulin resistance [9]. Adiponectin reduces glucose excretion from the liver and increases glucose uptake into muscle, preventing hyperglycemia [9]. Animal studies show that adiponectin increases insulin sensitivity and lowers glucose levels by improving insulin sensitivity. Adiponectin expression in adipose tissue and plasma concentration is depleted in obese and overweight people, and it’s unclear why [9]. This hormone reduces plasma-free fatty acids and increases oxidation. Adiponectin gene expression in culture medium decreases with fat mass and volume [10]. Keeping an eye on adiponectin levels in the plasma may be helpful in the management of metabolic syndrome patients who suffer from hypoadiponectinemia [11]. APQ AS, or adiponectin antisense lncRNA (long noncoding RNA), has been found to pair with adiponectin mRNA and reduce the translation of adiponectin, thereby reducing lipolysis through the negative regulation of adiponectin translation [12]. LncRNAs, which have more than 200 nucleotides, were first described in 1990 [13]. According to growing evidence, lncRNAs regulate cell development and differentiation, stem cell pluripotency, and human disease [14, 15]. LncRNAs, are a new type of RNA molecule that makes up the majority of the human transcriptome. LncRNAs have become some of the most important parts of key cellular pathways that control cellular proliferation, stem cell self-renewal, and reprogramming. Several types of human diseases have been linked to modifications in the expression of a large number of lncRNAs [16, 17]. Thus, impaired expression of this gene increases the likelihood of obesity [12, 18]. Recently, there has been tremendous focus on the possible role of metastasis-associated lung adenocarcinoma transcript 1 (MALAT1) in the pathogenesis of metabolic disorders [19].

Mets and glucose intolerance have low 25-hydroxyvitamin D [20]. 25-hydroxyvitamin D is inversely related to body mass index (BMI) [21], waist circumference (WC) [22, 23]. Numerous studies show conflicting effects of vitamin D on serum adiponectin levels. In a clinical trial, vitamin D intake did not affect adiponectin levels in diabetic patients [24]. In the Nurses’ Health Study (2012) cohort and the Health Professionals Follow-Up Study (2012), the results showed that an increase in serum vitamin D levels was directly related to serum adiponectin levels and therefore reduced cardiovascular disease [25]. Vitamin D modulates miR expression in fat cells in vitro and in vivo by affecting their signaling [26]. Recently, there has been tremendous focus on the possible role of MALAT1 in the pathogenesis of metabolic disorders and diabetic models [19, 27]. Vitamin D receptor acts as a regulator of MALAT1 gene expression [26, 28]. Vitamin D deficiency has been linked to elevated MALAT1 expression in patients with coronary heart disease, and vitamin D intake may be associated with modulation of this inflammatory lncRNA's expression in those patients [29].

The purpose of this study was to investigate the anti-inflammatory effects of vitamin D on serum adiponectin levels in light of the complicated link between vitamin D and inflammation in metabolic syndrome. Here, we looked at a possible change in the expression of lncRNAs MALAT1 and APQ AS that could be used for early diagnosis and prognosis.

## Methods

### Participants

This clinical trial included Mashhad University of Medical Sciences employees and students aged 30 to 50 who met the eligibility criteria. Participants were given either 200 mL of fortified milk (FM) or 200 mL of non-fortified milk (NFM) for ten weeks.

### Inclusion criteria

People interested in participating in the study completed the consent form. Other inclusion criteria are: waist circumference > 80 cm in women and > 94 cm in men; other criteria of the International Diabetes Federation [31], no specific underlying disease (such as malignancy, kidney or liver failure), Do not take medication that interferes with vitamin D (such as anticonvulsants, corticosteroids), vitamin D, or calcium supplements in the past three months. No smoking and no alcohol abuse. Pregnancy during the study, allergies or intolerance to dairy products, a new diagnosis of any disease, or starting any medication were all excluded. We got approval from the Ethics Committee of Mashhad University of Medical Sciences (IR.MUMS.MEDICAL.REC.1399.389) and registered the trial with the Iranian Clinical Trials Registry (IRCT20101130005280N27).

### Sample size and allocation

The number of participants in this study was estimated using a power analysis with an alpha of 0.05 and a beta of 20%; 36 individuals were counted for each group (based on Adiponectin) [30], but only 40 were used due to a 10% dropout. When assigning participants to the intervention and control groups, we used sealed envelopes labeled with either an A or B. Once the experiment was completed, researchers were given access to the allocation list, which had been kept secret by the Faculty of Medicine.

### Dietary intake and physical activity assessment

We asked the participants not to change their diet or consume any vitamin D supplements or fortified foods during the trial. Furthermore, to confirm no significant change in their diet, we documented their dietary patterns via 3-days food records at the beginning, middle, and end of the trial (2 business days and 1 day off). We classified and analyzed all food records by converting them to grams and using the Nutritionist IV software based on the US Department of Agriculture food composition table and applies to Iranian foods. The Beck physical activity questionnaire was applied to quantify the participants' physical activity [31]. For between-groups statistical analysis, the baseline values were adjusted.

### RNA extraction and cDNA synthesis

Total RNA was extracted from both frozen clinical samples using Trizol reagent (Invitrogen Life Technologies, Carlsbad, CA) and according to the manufacturer's instructions. The extracted RNA was treated with a DNase enzyme (Takara, Japan) in an RNase-free condition to remove any potential contamination with DNA molecules. Then, the first strand of complementary DNA (cDNA) was synthesized by using the Hyperscript RT reagent Kit (GeneAll, South Korea) and random hexamer primers (Takara, Japan), as described by the manufacturers. Table 1 contains a list of the primers used in this study.

**Table 1:** Verification of gene expression changes by qRT-PCR. Primers used in this paper

Transcript	Primer	Sequence (5ʹ → 3ʹ)	Lengths (bp)
APQ AS	F	CCCAGTTGGGACCTACAAAGG	21
	R	TTGGCAAGTCGACTCTTGGA	20
GAPDH	F	ATGGGGAAGGTGAAGGTCG	19
	R	GGGGTCATTGATGGCAACAATA	22
MALAT1	F	GAAGGAAGGAGCGCTAACGA	20
	M	TACCAACCACTCGCTTTCCC	20

### Statistical analysis

These efficiencies were used to adjust the real-time PCR results. All gene expression levels in clinical samples were standardized to the GAPDH gene, which served as an internal reference to calculate fold changes in gene expression. The expression of candidate genes in FM samples was normalized to that of matched NFM samples (2^-ΔΔCT method). Graphpad Prism was used to plot receiver operating characteristic (ROC) curve analysis to identify between FM and NFM clinical samples (version 8.0.2).

### Quantitative real-time PCR

Using the Gene Runner (version 3.05), PerIPrimer (version 1.1.21), and Oligo (version 7.56) software, specific PCR primers were designed (Table 2). Real Q plus 2 × master mix Green (Ampliqon, Denmark) supplemented with ROX dye was used for all quantitative real-time PCR reactions. As an endogenous control, the glyceraldehyde 3-phosphate dehydrogenase (GAPDH, NM_002046.4) transcript was quantified, and the expression of APQ AS and MALAT 1 expression was normalized to its expression level. Amplification has been completed for 40 cycles with denaturation at 95 °C for 15 secs, annealing and extending at 63 °C for 55 secs using the ABI STEP ONE real-time PCR system (Applied Biosystems, Foster City, CA). We also used melt curve analysis and direct sequencing to prove that the PCR products were real.

**Table 2:** Baseline characteristics of study population

Variables	FM group (n = 40)	NFM group (n = 39)	p-value^*^
Age (years)	43.47 ± 7.21	43.19 ± 7.21	0.82
Gender			
Male	19 (47.5%)	17 (43.5%)	0.45
Female	21 (52.5%)	22 (56.4%)	
Weight (kg)	76.22 ± 11.51	77.18 ± 11.94	0.78
BMI (kg/m^2^)	23.22 ± 2.81	23.49 ± 3.16	0.77
Waist circumference (cm)	94.29 ± 9.45	94.02 ± 9.72	0.83
Vitamin D (ng/mL)	14.08 ± 5.15	14.02 ± 5.16	0.94
Calcium (mg/dL)	9.33 ± 0.41	9.30 ± 0.43	0.69
Physical activity level	6.58 ± 1.49	6.56 ± 1.42	0.95

All values presented mean ± SD and percentage

^*^Using Student’s t-test

### Anthropometric indices

We used a wall stadiometer with a 0.1 cm accuracy to determine the height at baseline. A digital bio-impedance analyzer (TANITA BC 418) calculated weight and fat mass while wearing light shoes and clothes. The formula for assessing body mass index (BMI) was weight (kg)/height^2^ (m^2^). A single expert staff member measured the waist twice with a flexible tape at the midpoint between the lower edge of the ribs and the iliac crest at the end of a normal exhalation. WHR = WC (cm)/HC (cm). To measure the amount of adiponectin in the serum, Zelbio kits from Germany were used according to the manufacturer's instructions.

## Results

**Fig. 1:**
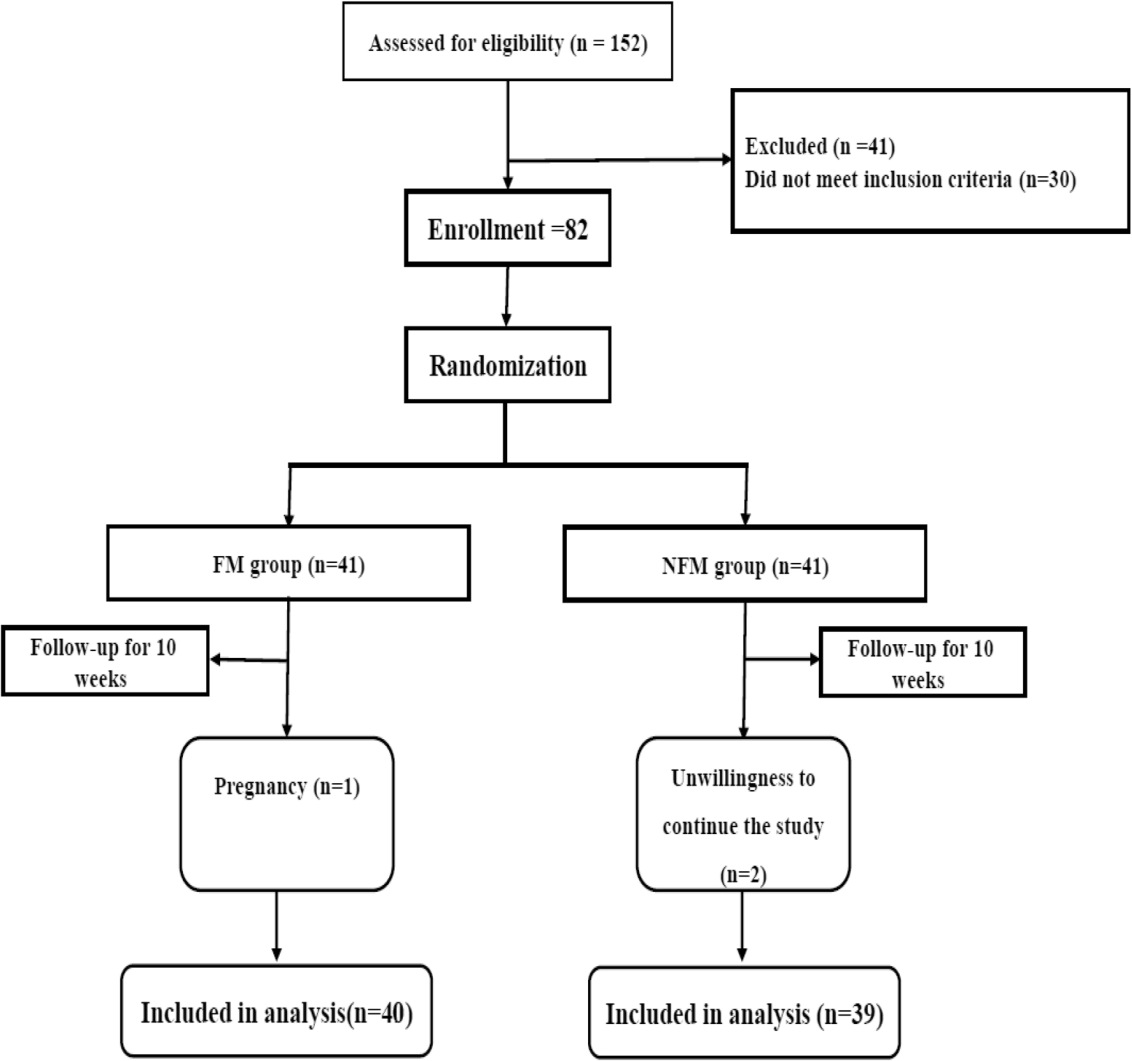
Flow diagram of study recruitment

**Fig. 2:**
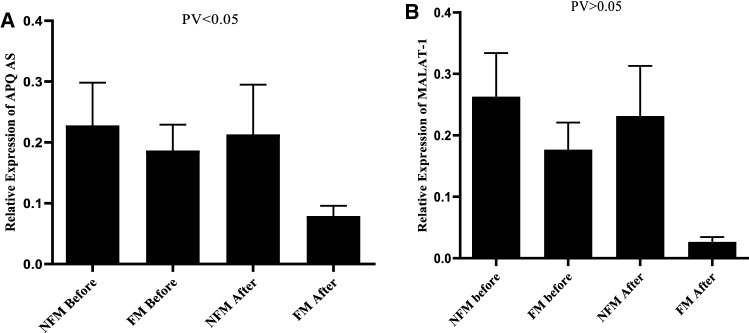
Differentially expressed lncRNAs APQ AS (**A**) and differentially expressed lncRNA MALAT1 (**B**)

**Fig. 3:**
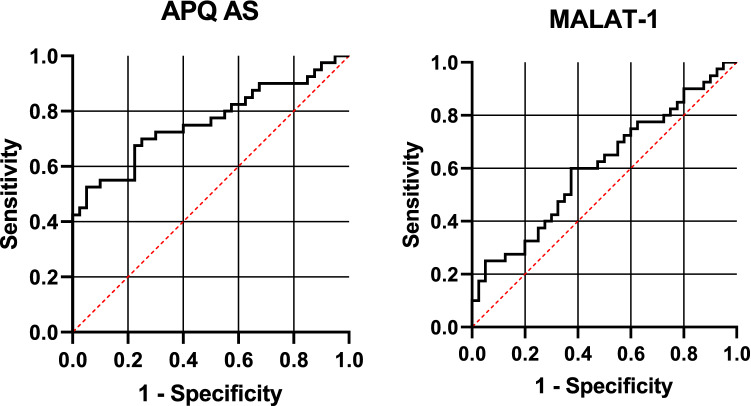
Analyzing the sensitivity and specificity of APQ AS [Area under the ROC curve 0.7625] and MALAT1 [Area under the ROC curve 0.6075]. The ROC curve analysis failed to discriminate between FM and NFM samples

In this study, 82 people met the eligibility criteria and were divided into fortified milk (FM) and non-fortified milk (NFM). Due to pregnancy, one participant in the FM group and two in the NFM group were excluded from the study (Fig. 1). The mean age in the FM and NFM groups was 43.47 ± 7.21 and 43.19 ± 7.21, respectively, and 47.5% were male, and 52.5% were female in the FM and NFM groups 43.5% were male and 56.4% of the participants were female (Table 1). At the start, the mean and standard deviation weight, BMI, and waist circumference were not significantly different between the two groups. As shown in Tables 2 and 3, the dietary intake, physical activity level, serum levels of vitamin D, and calcium in the two groups were compared between them. Serum adiponectin levels and 25 (OH) vitamin D in study participants are shown in Table 4 that there is no significant difference between the two groups in the baseline D. Nevertheless, after the intervention, serum adiponectin levels and 25 (OH) Vitamin D increased in both groups, which was significant in the FM group. To evaluate the expression of APQ AS in the serum samples of study participants, we used specific primers for each RNA transcript. Quantitative real-time PCR results show that downregulation occurred in the Mets samples in the baseline in both groups (Fig. 2). As shown in Fig. 3, there is no significant difference in APQ AS expression in the baseline between FM and NFM groups. We then examined the effect of vitamin D on APQ AS expression in these samples, which showed that vitamin D had downregulation in Mets samples, while APQ AS expression did not change in NFM samples (Fig. 3). Analyzing the sensitivity and specificity of APQ AS [Area under the ROC curve 0.7625] and MALAT1 [Area under the ROC curve 0.6075]. The ROC curve analysis failed to discriminate between FM and NFM samples.

**Table 3:** Dietary intakes of study population at the beginning and end of the intervention

Nutrients	FM group (n = 40)	NFM group (n = 39)	p-value^*^
Energy (kcal/day)			
Before	2132.25 ± 775.81	1994.03 ± 514.47	0.47
After	2246.47 ± 689.56	2063.29 ± 597. 31	0.69
p-value^#^	0.82	0.39	
Protein (g/day)			
Before	87.00 ± 17.92	84.32 ± 14.63	0.35
After	89.23 ± 19.03	86.50 ± 15.72	0.41
p-value^#^	0.33	0.17	
Carbohydrate (g/day)			
Before	325.99 ± 54.60	317.18 ± 46.27	0.6
After	327.42 ± 51.09	313.94 ± 48.42	0.47
p-value^#^	0.89	0.48	
Fat (g/day)			
Before	91.92 ± 14.64	83.79 ± 19.11	0.18
After	92.18 ± 12.22	86.31 ± 20.51	0.34
p-value^#^	0.57	0.09	

Data are expressed as mean ± standard deviation. p-value resulted from independent sample t test

^#^Resulted from paired sample t test

**Table 4:** Comparison of adiponectin FM group and NFM at baseline and after the intervention

Variables	FM group (n = 40)	NFM group (n = 39)	p-value^*^
Adiponectin (µg/liter)			
Before	101.5 ± 47.3	98.6 ± 45.8	
After	122.8 ± 59.3	105 ± 51.44	0.034
p-value^#^	0.01	0.09	
Vitamin D (ng/ml)			
Before	14.08 ± 5.15	14.02 ± 5.16	
After	19.1 ± 5.69	13.89 ± 5.85	< 0.001
p-value^#^	0.001	0.62	

Data are expressed as mean ± standard deviation

^#^Resulted from pair t-test

^*^P values based on ANCOVA after adjustment of baseline values

**Fig. 4:**
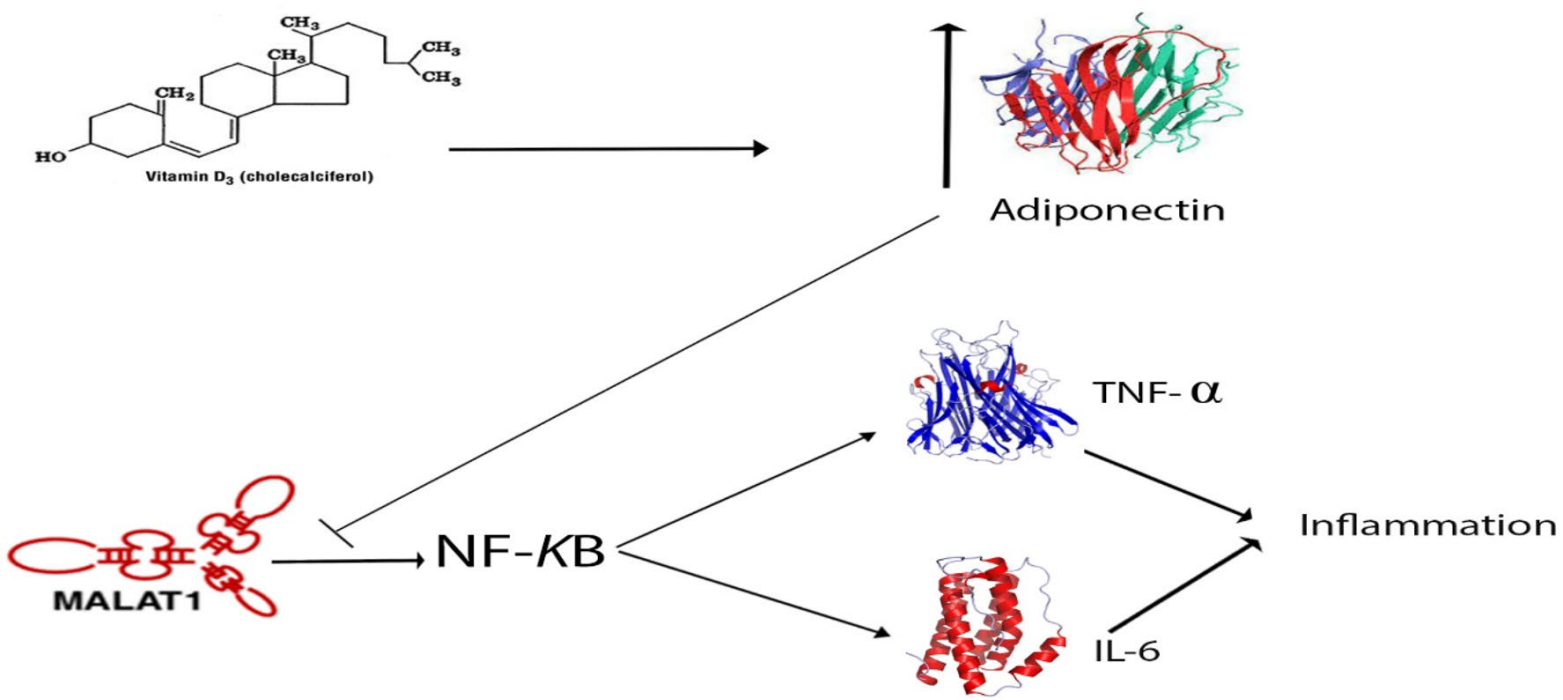
Vitamin D inhibits MALAT1 expression and reduces inflammation by increasing adiponectin

**Fig. 5:**
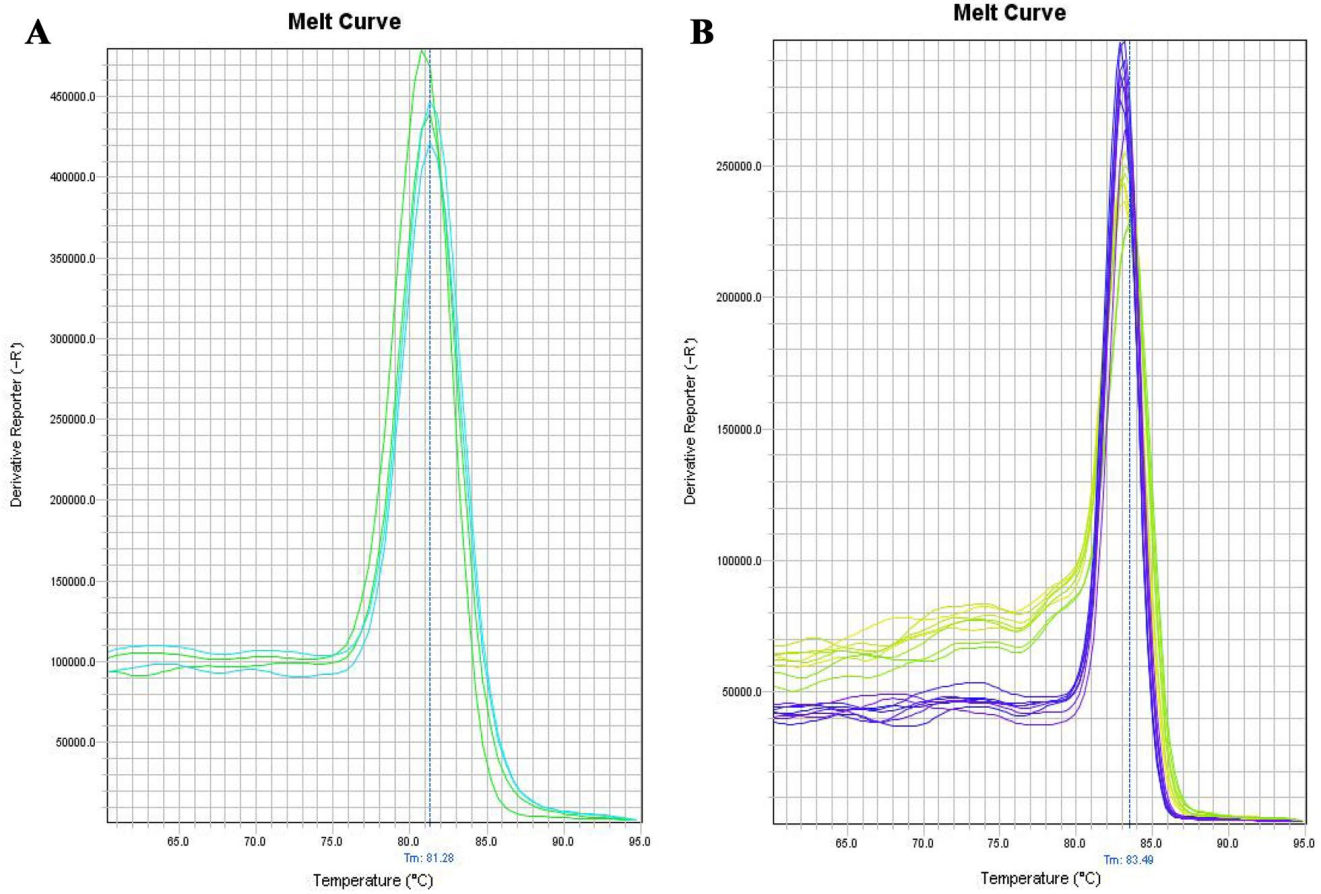
Melt curves of the APQ AS (**A**) and MALAT1 (**B**)

We also measured MALAT1 expression in Mets samples using a special primer for each transcript (Fig. 3B). MALAT1 expression decreases in the FM group, but not significantly between the two groups, according to quantitative real-time PCR.Vitamin D inhibits MALAT1 expression and reduces inflammation by increasing adiponectin (Fig. 4). According to melting temperatures curve, the presence of single peaks without additional peaks indicates the specificity and absence of primer dimer (Fig. 5).

## Discussion

The results of our study, which is the first to examine the effect of FM with 1500 IU of vitamin D on LncRNA gene expression in individuals with Mets, indicated that ten weeks of vitamin D FM was associated with increased serum adiponectin levels in individuals with Mets. Additionally, in individuals with Mets, the expression of LncRNAs APQ AS and MALAT1 was associated with downregulation in both groups, but the decrease in APQ AS was statistically significant.

These findings demonstrate that a therapeutic dose of vitamin D improves adiponectin. Vitamin D supplementation at 150,000 IU every three months failed to improve serum 25OH D or modulate inflammatory markers and adiponectin in obese and overweight adolescents [32]. 50,000 IU of vitamin D for eight weeks did not affect serum adiponectin levels in type 2 diabetics [33]. Due to the small sample size and lack of a control group in the first study, the second study did not reveal the positive effects of vitamin D on adiponectin gene expression. Vitamin D affects adiponectin in multiple ways. Renin-angiotensinogene regulates Adiponectin. Increased renin angiotensinogen activity causes abnormal adipocytes and less adiponectin. Vitamin D may boost adiponectin by reducing angiotensin production [25, 34]. Vitamin D acts as a negative regulator of renin expression and subsequent renin-angiotensin system activity [35, 36]. At the same time, adipocytes produce all components of the renin-angiotensin system of local adipose tissue that increase its activity inhibits the secretion of adiponectin [37, 38]. In addition, the activity of the renin-angiotensin system of adipose tissue increases with increased adipose tissue [39, 40]. Increased activity of the adrenal renin-angiotensin system may be a potential mechanism for the relative hypoadiponectinemia seen in obesity [34].

Insulin resistance and glucose intolerance are inflammatory syndromes linked to TNF-α, interleukin-1, and decreased adiponectin production. Vitamin D may increase serum adiponectin levels by lowering TNF-α gene expression. Vitamin D receptors on adipocytes showed a direct mechanism for vitamin D in adiponectin gene expression [41]. Vitamin D and calcium may regulate visceral adipose adipocytokine expression. In addition, osteocalcin stimulated adiponectin gene expression in adipocyte cell cultures [42]. Pre-diabetics’ serum adiponectin levels were unaffected by 2000 IU of vitamin D and 1200 mg of calcium carbonate for six months [43] and 4000 IU of vitamin D did not affect young obese people’s adiponectin for six months [44]. In diabetic elderly patients, a single oral dose of 300,000 IU of vitamin D did not significantly impact serum adiponectin levels [45]. Higher 25 (OH) D levels were associated with higher adiponectin levels in 1206 Nurses’ Health Study women and 439 Health Professionals Follow-Up Study men. Adiponectin and 25 (OH) D levels increase cardiometabolic disease risk [25]. A daily dose of 1000 IU of vitamin D for 12 months increased serum adiponectin in diabetic patients [24]. Hypoadiponectinemia is linked to endothelial dysfunction, increased intima-media thickness, and coronary artery calcification [46]. Increased circulating adiponectin levels during vitamin D supplementation could be a mechanism for improving arterial stiffness [47]. Recent research links vitamin D deficiency to hypoadiponectinemia [42, 44], so vitamin D supplementation may enhance adiponectin levels. Obesity may benefit from this finding because a lack of adiponectin and vitamin D has been linked to the progression of obesity [48–49].

A decrease in APQ AS gene expression was observed in both groups but was more pronounced in the FM group. APQ AS is more stable than adiponectin mRNA [12]. Adiponectin translation is reduced by APQ AS, which is expressed in adipocytes and paired with Adiponectin mRNA [12]. Thus, any APQ AS lncRNA disruption may increase obesity risk [12]. Vitamin D’s effect on these lncRNAs has been contradictory due to the decrease in MALAT1 expression in the FM group. MALAT1 expression has been associated with increased oxidative stress and pro-inflammatory cytokines in diabetic and non-alcoholic fatty liver disease (NAFLD) models [35–37]. The literature on the role of MALAT1 in obesity and metabolic syndrome is limited and conflicting [50], but MALAT1 was recently found to be reduced in white adipose tissue from obese mice. However, its deletion had no stimulatory or inhibitory effects on diet-induced adipose tissue gain and lipid homeostasis in obese mice [37], and The mechanism of MALAT1 in inflammation related to vitamin D remains unknown [34]. Vitamin D deficiency has been linked to increased MALAT1 expression in patients with coronary heart disease, and vitamin D intake may be associated with modulation of the expression of this inflammatory lncRNA in those patients [29].

Our study has several limitations that measuring total adiponectin levels can be a limitation. More extensive studies are needed to establish the beneficial vascular effect of vitamin D fortified milk and the clinical effect on cardiovascular outcomes in people with metabolic syndrome. In conclusion, serum adiponectin levels in Mets were shown to increase after 10 weeks of vitamin D FM supplementation. Expression of both MALAT1 and APQ AS was correlated with downregulation, but only APQ AS reached statistical significance in Mets. In these cases, a therapeutic amount of vitamin D was found to increase levels of adiponectin.

## Data Availability

The datasets used and/or analyzed during the current study are available from the corresponding author on reasonable request.
